# Abstract withdrawn by the author

**DOI:** 10.1186/1532-429X-15-S1-P189

**Published:** 2013-01-30

## Abstract withdrawn by the author

